# Optimizing the management of electrophysiology labs in Chinese hospitals using a discrete event simulation tool

**DOI:** 10.1186/s12913-024-10548-5

**Published:** 2024-01-12

**Authors:** Wenjuan Lin, Lin Zhang, Shuqing Wu, Fang Yang, Yueqing Zhang, Xiaoying Xu, Fei Zhu, Zhen Fei, Lihua Shentu, Yi Han

**Affiliations:** 1 Department of Nursing, The First Affiliated Hospital, Zhejiang University School of Medicine, Hangzhou, Zhejiang Province China; 2 Sun Yat-Sen Memorial Hospital of Sun Yat-Sen University, Guangzhou, Guangdong Province China; 3 Health Economic Research Institute, Sun Yat-sen University, 132 East Waihuan Road, Guangzhou, Guangdong Province 510006 PR China

**Keywords:** Cardiac arrhythmias, Atrial fibrillation, Electrophysiology, Discrete event simulation, Operations research, Operating room

## Abstract

**Background:**

The growing demand for electrophysiology (EP) treatment in China presents a challenge for current EP care delivery systems. This study constructed a discrete event simulation (DES) model of an inpatient EP care delivery process, simulating a generalized inpatient journey of EP patients from admission to discharge in the cardiology department of a tertiary hospital in China. The model shows how many more patients the system can serve under different resource constraints by optimizing various phases of the care delivery process.

**Methods:**

Model inputs were based on and validated using real-world data, simulating the scheduling of limited resources among competing demands from different patient types. The patient stay consists of three stages, namely: the pre-operative stay, the EP procedure, and the post-operative stay. The model outcome was the total number of discharges during the simulation period. The scenario analysis presented in this paper covers two capacity-limiting scenarios (CLS): (1) fully occupied ward beds and (2) fully occupied electrophysiology laboratories (EP labs). Within each CLS, we investigated potential throughput when the length of stay or operative time was reduced by 10%, 20%, and 30%. The reductions were applied to patients with atrial fibrillation, the most common indication accounting for almost 30% of patients.

**Results:**

Model validation showed simulation results approximated actual data (137.2 discharges calculated vs. 137 observed). With fully occupied wards, reducing pre- and/or post-operative stay time resulted in a 1–7% increased throughput. With fully occupied EP labs, reduced operative time increased throughput by 3–12%.

**Conclusions:**

Model validation and scenario analyses demonstrated that the DES model reliably reflects the EP care delivery process. Simulations identified which phases of the process should be optimized under different resource constraints, and the expected increases in patients served.

## Background

Cardiac arrhythmias encompass a wide range of heart rhythm and heart rate disorders [1]. Clinical presentation can range from asymptomatic to life-threatening, such as sudden cardiac arrest [1]. Arrhythmias can be managed with antiarrhythmic medication or various electrophysiological (EP) procedures, such as ablation or implantation of pacemakers or defibrillators [2]. The most common type of arrhythmia is atrial fibrillation (AF) [3], which is linked to higher risks of stroke, heart failure, dementia, and death [4–9]. This significantly impacts healthcare costs owing to hospitalizations and loss of productivity [10].

The prevalence of AF in China is estimated to be 1.6% and increases with age [11]. This poses a formidable challenge to China’s healthcare system owing to its aging population [12]. While the ablation treatment rate among Chinese AF patients is unclear, it is reasonable to assume that over 176,000 ablation procedures may be needed based on the US ablation rate of 0.79% in 2005 [13].

Furthermore, overcrowding at tertiary public hospitals is a major problem in China [14]. While economic growth slows, rapid expansion of hospital operations and infrastructure is not a viable option to meet the demand for EP treatments [15]. Thus, decision-makers must utilize limited medical resources to deliver healthcare services more efficiently [15] in order to meet this growing demand.

To this end, China’s National Health Commission has issued guidelines to improve public hospital operational management and healthcare delivery [16]. The Commission not only recommends optimizing resource allocation and processes but also emphasizes data collection and analysis, as well as improving the quality of decision-making [16]. Public hospitals are urged to collect and manage operational data for analysis, establish an analytical decision-making framework, and implement analysis results [16].

Optimizing EP service delivery efficiency is a complex, multidimensional problem involving factors ranging from patient flow management to novel technology adoption. Discrete event simulation (DES) is an operational research tool that helps decision-makers assess different management strategies, enabling them to evaluate not only the performance of current healthcare delivery systems but also that of hypothetical scenarios [17]. This allows them to choose between different approaches of healthcare delivery to prioritize and pursue without undesirably impacting current systems [17].

This study established a generalized DES model of an inpatient EP treatment process, which can be applied across tertiary hospitals in China. The model examines how cardiology departments under different resource constraints can serve more EP patients by improving the efficiency of each phase of the healthcare delivery process, including pre-operative preparation, operative time, or post-operative recovery. This enables hospital decision-makers to clarify which phases of the EP care delivery process to prioritize in different situations in order to better meet the demand for EP treatment.

## Methods

### Model setting and perspective

We constructed a model to simulate the inpatient journey of individual EP patients from admission to discharge in the cardiology department of a tertiary hospital in China, using the “simmer” package [18] in R [19]. To build a generalized model applicable to different hospitals in China, clinicians from two large tertiary hospitals were consulted. We established a preliminary design for the model based on the inpatient management process of the Cardiology Department of the First Affiliated Hospital of Zhejiang University (FAHZU). We further examined the patient care delivery flow at Shanghai General Hospital’s Cardiology Department, fine-tuning the preliminary design. The final model abstracted and generalized a common inpatient care pathway without focusing on implementation details specific to an individual hospital.

These two cardiology departments have a number of catheter laboratories, some of which are dedicated electrophysiology laboratories (EP labs) that are prioritized for EP procedures. They can only be used for other cardiology procedures after all EP procedures scheduled for that day are completed.

The primary study population is EP patients who pass through the EP labs (i.e., the target EP patients, TEP patients). The model explicitly tracks the pre-operative phase, operation, and post-operative stay of these patients. The other two patient types include catheter lab EP patients (CLEP patients) who receive their procedure in the catheter labs and non-EP cardiology patients (NEP patients). Both CLEP and NEP patients compete with TEP patients for hospitalization resources, but not for resources used in EP lab operations. Therefore, only the hospitalization stays of CLEP and NEP patients were included in the model.

### Model structure

#### Arrival process

The model generates three patient types: TEP, CLEP, and NEP. Unlike patients receiving care in outpatient facilities or emergency rooms, inpatient visits for elective procedures do not arrive randomly at the hospital. Patients in this model are scheduled to arrive at the same time daily (one hour before working hours). The daily arrivals of each type of patient was randomly generated.

#### Resources

The model considers three resource types: cardiology ward beds, electrophysiologists, and EP labs. All three types of patients share bed resources, but only TEP patients require electrophysiologists and EP labs. The schedules for electrophysiologists and EP labs are managed by hospital staff.

#### Inpatient process

**Fig. 1:**
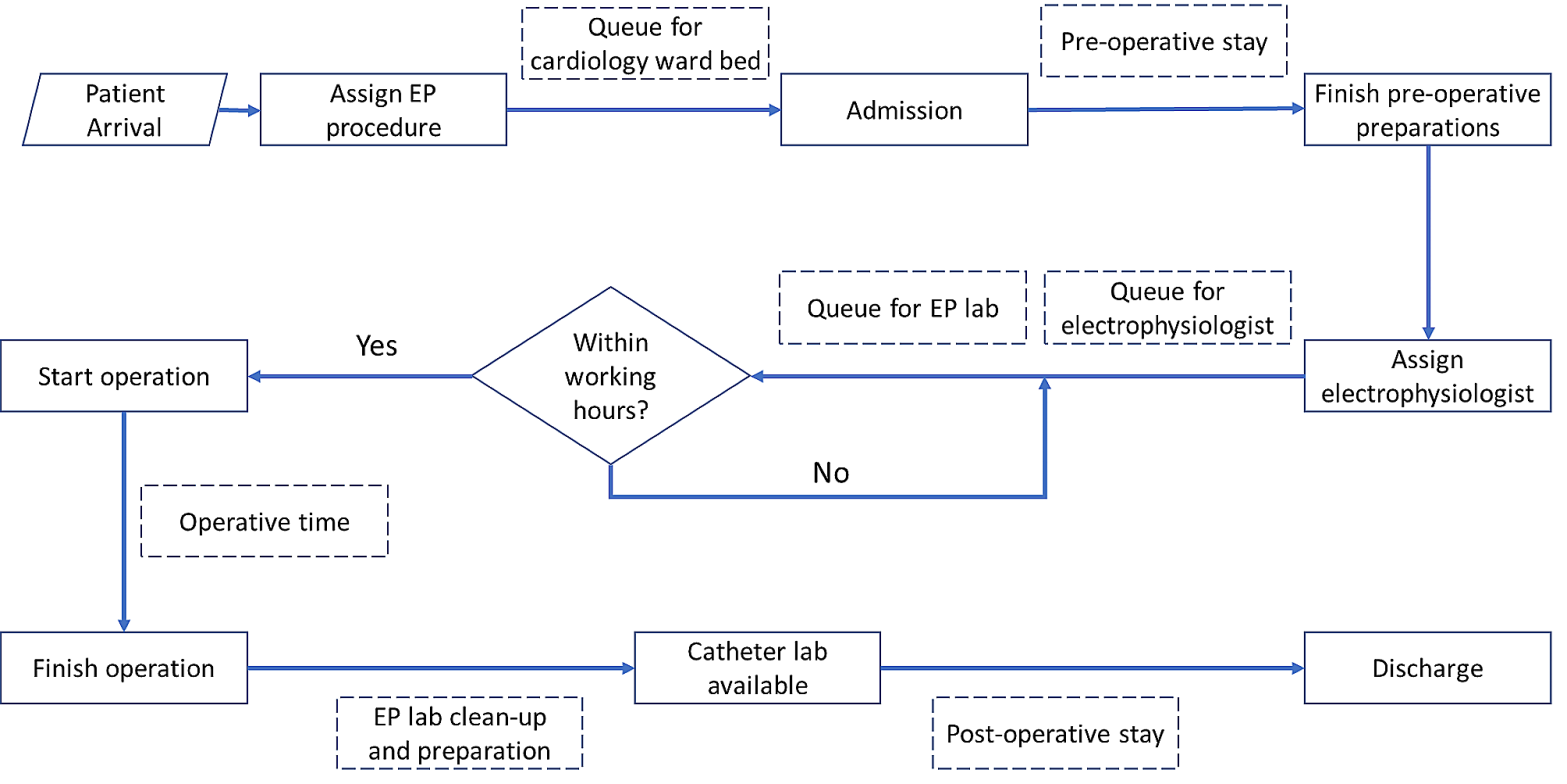
Model process

The process for TEP patients is shown in Fig. 1. Upon arrival, the TEP patient is assigned to one of several EP procedures and admitted if a ward bed is available. The patient’s stay consists of three phases: pre-operative stay, EP procedure, and post-operative stay. Once the patient’s pre-operative phase is complete, they are assigned to an electrophysiologist and enter the queue for EP lab procedures the next day. If the EP labs are not open the next day, the patient waits until the subsequent workday. Once the patient’s assigned electrophysiologist and an EP lab become available during working hours, the patient enters the EP lab to undergo the procedure. Once a procedure is started, it will continue until completion, regardless of the pre-defined working hours. However, no new procedures are allowed after working hours. Upon procedure completion, the electrophysiologist leaves the EP lab and the patient returns to the ward. The catheter lab becomes available after cleanup and preparation for the next procedure. After post-operative recovery, the patient is discharged.

For CLEP and NEP patients, the process is simpler. Patients are admitted if ward beds are available and discharged after their length of stay is reached.

#### Model inputs

Model inputs were based on actual data collected between May 1–June 30, 2022 from FAHZU’s Cardiology Department. Admission and discharge dates were recorded for TEP, CLEP, and NEP patients. Inputs related to the number of admissions per day and length of stay were based on patients admitted between May 1–June 25, as both admission and discharge dates were available for these patients. In addition, for TEP patients, procedure dates, procedure types, procedure start and end times, and assigned electrophysiologists were also collected.

#### Simulation

The daily arrival of TEP, CLEP, and NEP patients is modeled according to a negative binomial distribution. The procedure type for TEP patients is randomly drawn according to the proportions of the different EP procedures. Their electrophysiologist is randomly assigned based on the day of the week of their procedure and the average allocation of patients between different electrophysiologists on that day.

Processing times of TEP patients are determined by procedure type. The length of stay and days of pre-operative stay are randomly generated from a truncated lognormal distribution and rounded to integers. The post-operative stay is the difference between the length of stay and the pre-operative stay. The operative time, in minutes, is also modeled by a truncated lognormal distribution.

Following ISPOR guidelines, the simulation first runs for ten days with CLEP and NEP patients as a “warm up” period [20]. The TEP patient flow is generated from day 11. The simulation is then run for an additional 61 days, corresponding to the observation period of the actual data. Each scenario was simulated 1000 times.

#### Validation

The simulated total number of discharged TEP patients per replication and the simulated daily discharges were compared against actual data. Means and standard deviations are reported. The simulated and real daily discharges were also compared using the Mann-Whitney U test.

#### Scenario analyses

There are two significant capacity-limiting scenarios (CLS) for hospital cardiology departments in China. First, cardiology departments often must manage inpatient beds as a shared resource. Therefore, in this CLS, the inpatient ward is fully occupied before the EP labs reach their capacity. Second, EP lab capacity may be overwhelmed by demand. The second CLS usually occurs in hospitals with insufficient EP labs compared to ward beds.

Based on data from FAHZU, we performed scenario analyses to first investigate the patient throughput capacities under both CLSs and then the potential throughput when different phases of the inpatient process were optimized. Since optimizing all procedure types simultaneously in the real world is difficult, AF ablations (for paroxysmal and persistent AF) were selected for scenario analysis, as these were the most common procedures accounting for almost 30% of TEP patients. Patient throughput was measured as the total number of discharged TEP patients. For each CLS, we report the mean and standard deviation of the results from 1000 simulations.

First, we examined the scenarios with fully occupied cardiology ward beds. Because input from FAHZU had higher bed utilization than that of EP labs, changing the number of resources was not necessary. We increased the number of TEP patients arriving daily so that they would fully occupy any available beds remaining after the other two types of patients were admitted. This was considered the base case scenario under the condition of a fully occupied cardiology ward. Under this scenario, we reduced total length of stay of paroxysmal and persistent AF ablation patients by 10%, 20%, and 30% as the test cases. For each level of reduction, we calculated the expected difference in days and applied this reduction to different phases: the pre-operative stay (the minimum pre-operative stay remained 1 day); the post-operative stay; and both the pre-operative and post-operative stays reduced proportionally (the minimum pre-operative stay remained 1 day). For comprehensiveness, we also examined a scenario where operative times of paroxysmal and persistent AF ablation were reduced by 10%, 20%, and 30%, although this was expected to result in minimal change while EP labs were not at capacity.

We then analyzed scenarios with fully occupied EP labs. In addition to adjusting the patient flow as described above, the number of ward beds and EP lab resources were adjusted. This was the base case scenario under the condition of fully occupied EP labs. For comparison, we generated scenarios with reduced length of stay and reduced operative time in the same manner as above.

## Results

### Model inputs

FAHZU’s cardiology department has 87 ward beds, two EP labs, and eight electrophysiologists. Two EP labs are available every workday (Monday to Friday) from 8:00 AM to 10:00 PM. Two electrophysiologists are on duty during the working hours of each workday. Average patient allocation between the two electrophysiologists on duty each day is shown in Table 1.

**Table 1:** Division of patients between 2 electrophysiologists each weekday

Weekday	Proportions
Monday	72.7% / 27.3%
Tuesday	18.4% / 81.6%
Wednesday	50.0% / 50.0%
Thursday	53.2% / 46.8%
Friday	29.2% / 70.8%

The number of daily arrivals by patient type is shown in Table 2. Inputs relating to processing times for each phase in the TEP patient’s journey are summarized in Tables.

**Table 2:** Daily arrivals by patient type

Patient type	Mean (SD)
TEP patients	2.446 (2.358)
CLEP patients	3.875 (2.684)
NEP patients	13.161 (5.990)

Table 3, stratified by EP procedure type. Average length of stay for CLEP and NEP patients was 5.427 (SD 4.261) and 3.758 (SD 2.718), respectively.

**Table 3:** Model inputs of TEP patients by EP procedure type

EP procedure type	Proportion	Operative time, minutes		EP lab clean-up and preparation, minutes		Length of stay, days		Pre-operative stay, days	
		Mean (SD)	Min-Max	Mean (SD)	Min-Max	Mean (SD)	Min-Max	Mean (SD)	Min-Max
Paroxysmal AF ablation	18.4%	204.947 (66.735)	65–340	7.467 (7.477)	2–40	5.050 (2.350)	3–11	2.000 (2.151)	1–8
Persistent AF ablation	9.6%	208.688 (51.927)	128–321	5.800 (2.833)	2–11	3.375 (1.061)	3–6	1.375 (1.061)	1–4
PVC ablation	16.2%	107.206 (40.630)	35–202	6.963 (4.485)	1–25	2.810 (1.209)	2–7	1.400 (0.681)	0–3
SVT ablation	17.6%	99.067 (30.770)	70–209	5.130 (2.117)	1–10	2.478 (0.730)	1–4	1.250 (0.550)	0–2
AFlutter/PAC/AT ablation	5.9%	212.545 (149.095)	70–574	9.286 (9.032)	1–28	4.000 (2.646)	2–9	1.333 (0.816)	1–3
GP ablation	2.2%	99.000 (52.849)	50–155	6.500 (2.121)	5–8	2.500 (0.707)	2–3	2.000 (0.000)	2–2
PFO closure	2.2%	35.333 (6.110)	30–42	7.500 (3.536)	5–10	3.000 (0.000)	3–3	1.000 (0.000)	1–1
ICD implant	1.5%	99.250 (41.979)	65–160	6.667 (2.887)	5–10	5.000 (0.000)	5–5	1.000 (0.000)	1–1
Dual-chamber PM implant	5.9%	104.063 (31.693)	49–167	8.444 (4.773)	2–15	4.500 (1.732)	3–7	2.000 (1.414)	1–3
CRT PM implant	3.7%	190.200 (67.087)	94–267	5.600 (3.362)	2–10	6.600 (3.715)	4–12	2.200 (2.168)	1–6
Miscellaneous EP procedures	16.9%	86.741 (54.488)	27–305	6.600 (4.619)	2–25	2.917 (1.165)	2–5	1.167 (0.577)	1–3

PVC, premature ventricular complex; SVT, supraventricular tachycardia; AFlutter, atrial flutter; PAC, premature atrial contraction; AT, atrial tachycardia; GP, ganglionated plexi; PFO, patent foramen ovale; ICD, implantable cardioverter defibrillator; PM, pacemaker; CRT, cardiac resynchronization therapy

### Model validation

**Fig. 2:**
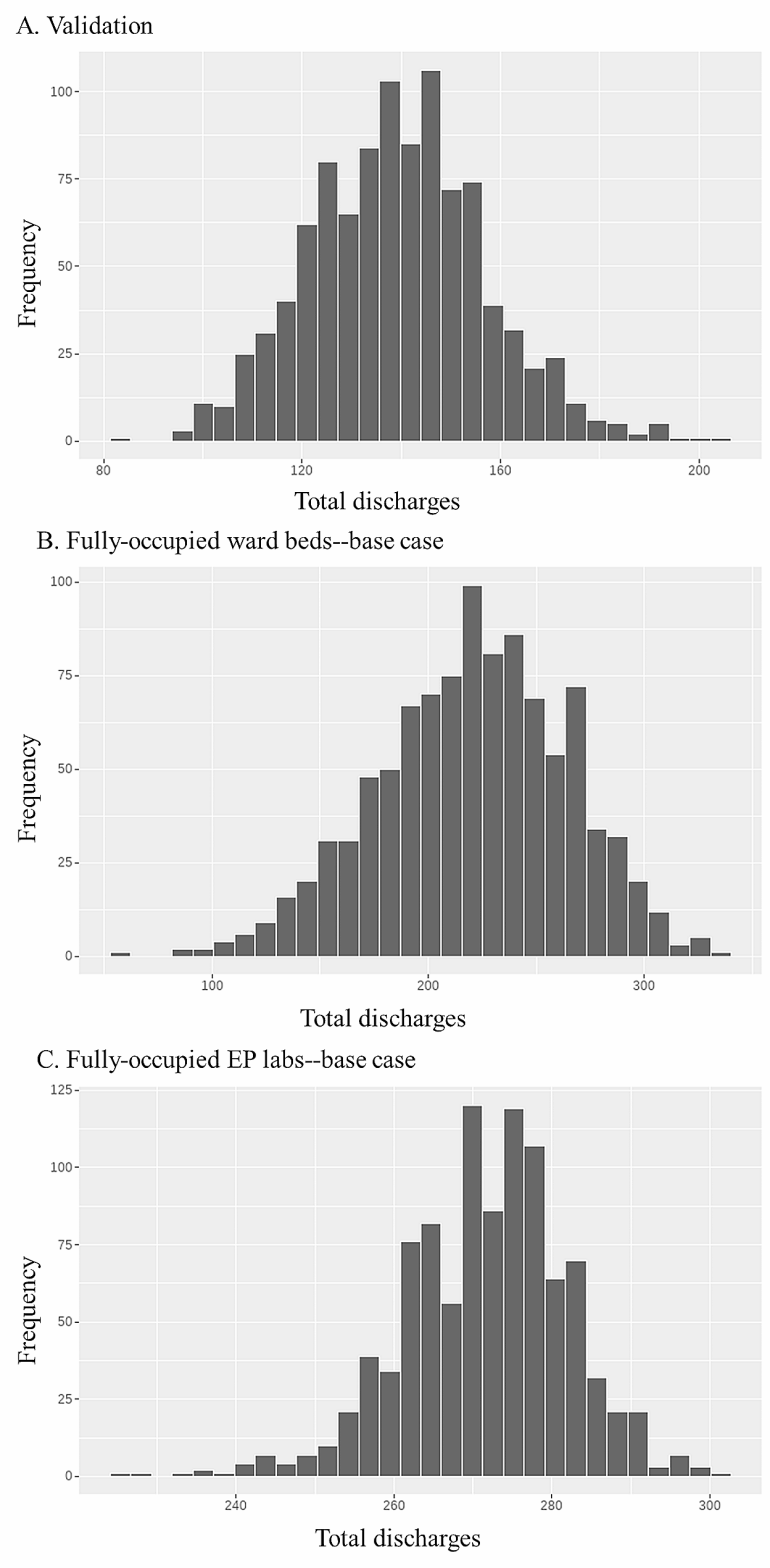
Distributions of total TEP patients discharged per replication

The simulated total number of TEP patients discharged per replication was 137.167 (SD 17.856), compared to 137 in the actual data. The distribution of the simulated results is shown in Fig. 2A.

**Fig. 3:**
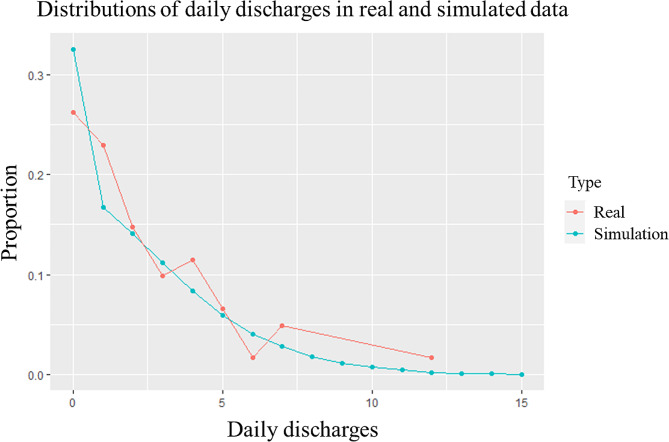
Model validation-distributions of real and simulated daily discharges

The simulated daily number of discharges was 2.249 (SD 2.455), compared with the actual figure of 2.245 (SD 2.364). Distribution of the simulated results, compared with that of actual results, is shown in Fig. 3. The Mann-Whitney U test showed no significant difference between the locations of the two distributions (*p* = 0.747).

### Scenario analysis

**Fig. 4:**
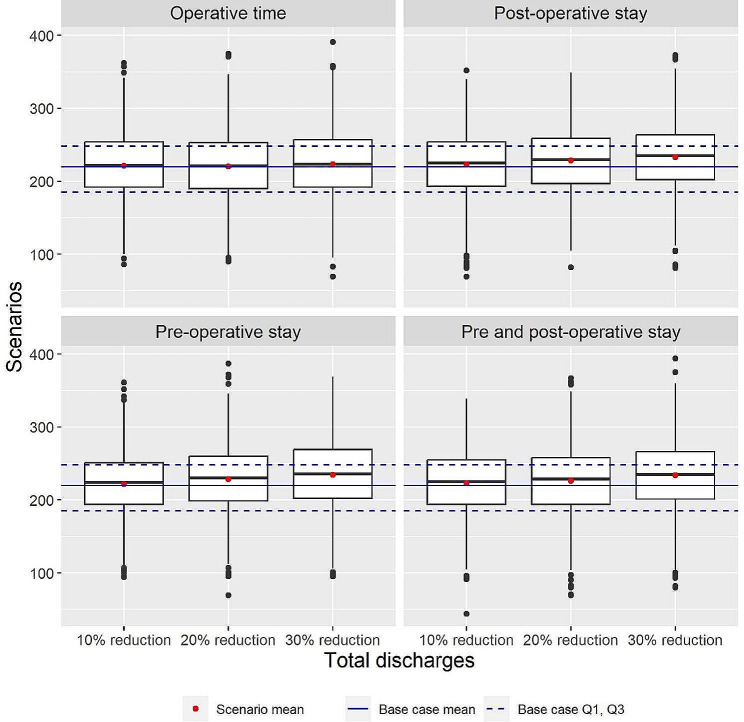
Boxplots of total discharges per replication with fully occupied ward beds

In the base case scenario with fully occupied cardiology wards, the total number of discharges per replication was 220.612 (SD 44.110), the distribution of which is shown in Fig. 2B. The results of this group of scenarios are presented in Table 4; Fig. 4. Paroxysmal and persistent AF ablation patients, whose processing times are adjusted in these scenarios, comprise approximately 30% of all patients. Reducing their length of stay increased the total number of discharges by 1–7%, regardless of which phase the reduction was applied to. Reducing operative time did not have any apparent effect.

**Table 4:** Total TEP patients discharged per replication with fully occupied ward beds

Scenario	Mean (SD)	Percent difference*
Base case	220.612 (44.110)	--
Reduce operative time		
By 10%	218.594 (45.473)	-0.9%
By 20%	219.996 (46.620)	-0.3%
By 30%	222.142 (46.260)	0.7%
Reduce pre-operative stay		
By 10% of length of stay	223.433 (46.153)	1.3%
By 20% of length of stay	225.816 (45.295)	2.4%
By 30% of length of stay	234.709 (47.664)	6.4%
Reduce post-operative stay		
By 10% of length of stay	222.007 (45.804)	0.6%
By 20% of length of stay	228.884 (45.141)	3.7%
By 30% of length of stay	232.116 (45.233)	5.2%
Reduce pre- and post-operative stay		
By 10% of length of stay	221.757 (44.698)	0.5%
By 20% of length of stay	228.324 (47.395)	3.5%
By 30% of length of stay	236.724 (47.252)	7.3%

*Relative to base case scenario

**Fig. 5:**
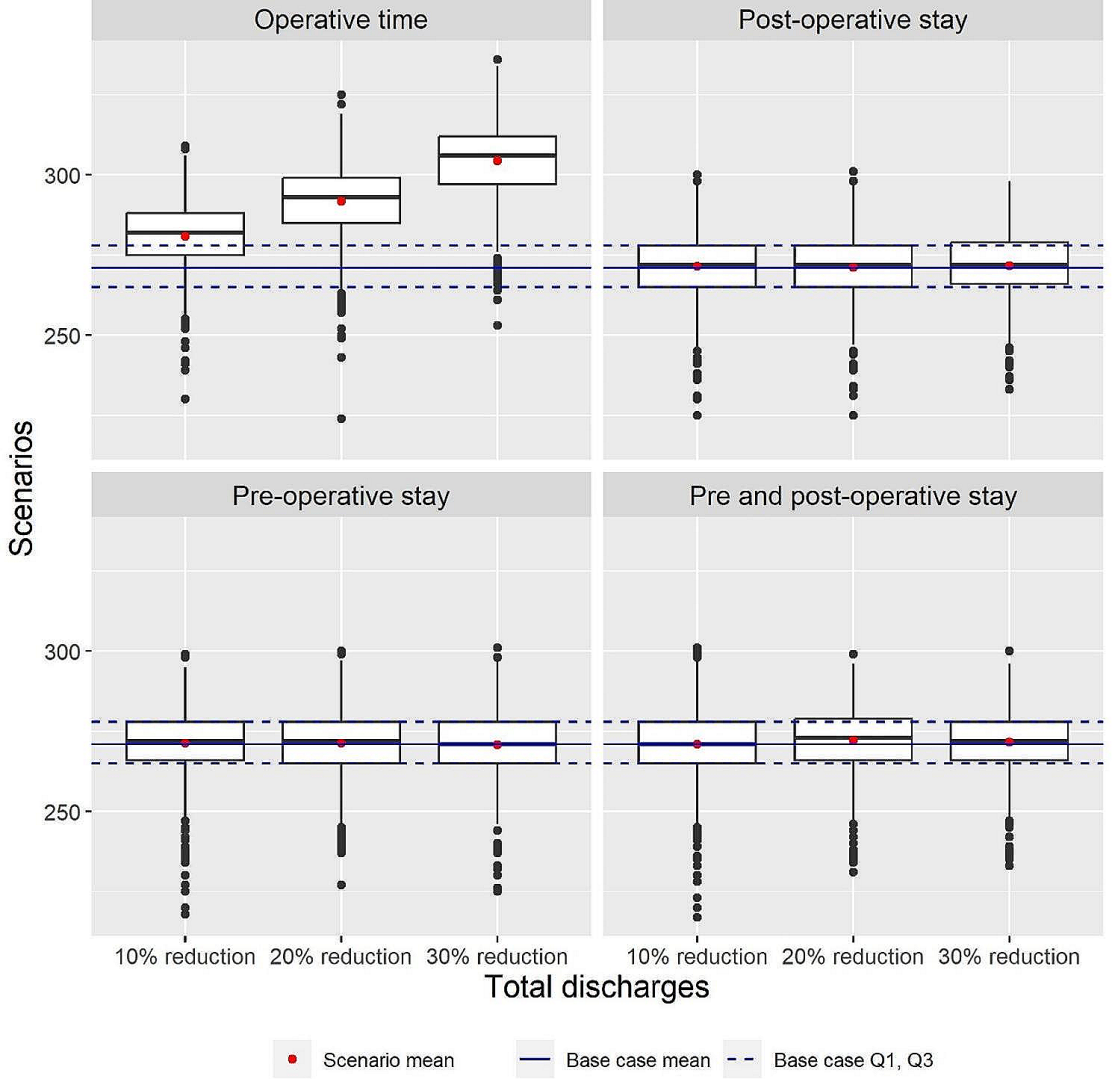
Boxplots of total discharges per replication with fully occupied ward beds

To simulate conditions with fully occupied EP labs, the number of ward beds was increased to 110 and the number of EP labs was decreased to one. The results of these scenarios are shown in Table 5; Fig. 5. Average total discharges was 271.634 (SD 10.379) in the base case scenario. The distribution is shown in Fig. 2C. Reducing operative time increased the total number of discharges by 3–12%. A reduction in the length of stay did not affect the total number of discharges.

**Table 5:** Total TEP patients discharged per replication with fully occupied EP labs

Scenario	Mean (SD)		Percent difference*
Base case	271.634 (10.379)		--
Reduce operative time			
By 10%	281.774 (10.519)		3.7%
By 20%	292.445 (11.163)		7.7%
By 30%	304.253 (12.181)		12.0%
Reduce pre-operative stay			
By 10% of length of stay	271.300 (10.546)		-0.1%
By 20% of length of stay	271.297 (9.931)		-0.1%
By 30% of length of stay	271.301 (9.688)		-0.1%
Reduce post-operative stay			
By 10% of length of stay	271.207 (10.229)		-0.2%
By 20% of length of stay	271.297 (10.512)		-0.1%
By 30% of length of stay	271.643 (10.205)		0.0%
Reduce pre- and post-operative stay			
By 10% of length of stay	271.225 (10.439)		-0.2%
By 20% of length of stay	271.434 (10.353)		-0.1%
By 30% of length of stay	271.213 (9.380)		-0.2%

*Relative to base case scenario

## Discussion

In this study, we developed and validated a generalizable DES model based on inpatient EP care delivery processes from two large tertiary hospitals in China. We used the model to simulate hospitalization stays of EP patients in scenarios with fully occupied ward beds and EP labs, and evaluated the effects of accelerating different phases of the delivery process under the two conditions. The model can support hospital decision-makers to identify which phase of the EP care delivery process to prioritize under different resource constraints in order to best satisfy demand for EP treatment. Decision-makers can then consider different methods of EP care delivery to target the relevant phase.

Under the condition of a fully occupied ward, reducing operative time had little to no effect, as this was not the bottleneck. On the other hand, reducing length of stay by 10–30% in paroxysmal and persistent AF ablation patients, which account for approximately 30% of all patients, increased total discharges by 1–7%. There was no difference whether the reduction was applied in the pre-operative or post-operative phase. However, it is imperative that reduction in hospitalization time does not compromise the quality of care. This goal can be achieved by integrating evidence-supported new technology or practices. For instance, the prevailing practice in China involves a pre-operative transesophageal echocardiogram (TEE), typically necessitating a hospital stay of 2 to 3 days prior to the procedure [21]. Intracardiac echocardiography (ICE) has been demonstrated as a safe and effective alternative to TEE, conducted during the procedure, thereby requiring only a 0–1 day pre-operative stay [21]. Pertaining to the post-operative phase, proactive prevention of complications contributes to reducing the length of stay while maintaining the quality of care [22]. This can be realized through careful management of patients with pre-existing medical conditions [23] and the accumulation of physician experience [24]. However, in the context of large tertiary hospitals in China, as in our study, the complication rate is generally well managed, leaving limited room for further improvement. In addition, the post-operative stay in China exceeds that of the US, where the standard practice leans towards an overnight stay [25], or even outpatient procedures in some instances [22]. This disparity may be attributed to the US’s adoption of diagnostic-related groups (DRGs) [26]. It is expected that the length of stay in China may decrease as the DRG payment system gains traction in the Chinese healthcare landscape [27].

When EP labs were fully occupied, reducing operative time by 10–30% in AF ablation patients led to a 3–12% increase in total discharges This improvement is made possible by several technological innovations that can reduce AF ablation operative times. The key to AF ablation is achieving pulmonary vein isolation [28]. Conventional point-to-point radiofrequency (RF) ablation involves creating multiple lesions, which may leave gaps in between [29] and drive long operative times [30]. Better navigation techniques, such as remote magnetic navigation-guided RF ablation, may reduce operative times [31]. The Q-FFICIENCY trial showed that very high-power, short-duration ablation using contact force-sensing RF catheters can reduce operative times by almost 50% [32], which is higher than the 30% reduction used in our analyses. According to the model, this reduction level would result in 330 total discharges (21% increase) using the specifications described previously.

In simulations of fully utilized EP labs, reducing operative time led to a more significant increase in discharges compared to reducing the length of stay in scenarios with fully occupied ward beds. This discrepancy may stem from the fact that the existing length of stay is already brief, allowing limited room for reduction, while there is more potential for improving EP lab efficiency. Our real-world data revealed that, over a two-month period, on 25 out of 40 working days, the working hours in the EP labs exceeded 8 h, with 29 days surpassing 7 h. This underscores the high utilization rate of the EP labs on a substantial number of days. However, it’s essential to note that reducing operative time does not necessarily ensure improved throughput, given the inherent variability in operational processes, including fluctuating patient flows. This variability in real-world settings emphasizes the need for adaptable and versatile modeling approaches. Our model aims not only to analyze the specific hospital from which the data originated but also to demonstrate its broader applicability as a decision-making tool for hospital managers. Recognizing the variability in hospital conditions, we tested two scenarios reflecting significant operational challenges, particularly relevant in China. These scenarios illustrate how our model can adapt to different settings, providing insights into potential operational improvements. The high utilization of the EP labs observed in our data exemplifies one of these challenges, highlighting the necessity for contextually informed strategies to enhance hospital efficiency.

While US practices and technologies provide useful insights as described above, it is crucial to tailor these approaches to align with China’s unique healthcare landscape. For instance, the adaptation from the DRG system to the diagnosis-intervention packet (DIP) system in China arose from challenges faced during a DRG pilot program [33]. The DIP system offered a less technically demanding and more scalable approach [33]. Since 2020, China has been piloting a dual-track arrangement with both DRGs and DIP, acknowledging the diverse regional capacities of local health systems [33]. This initiative underlines the importance of contextualizing global practices to improve resource allocation and potentially reduce hospital stay lengths without compromising care quality, aligning with the overarching objective of enhancing efficiency in healthcare delivery.

A previous study by Kowalski et al. investigated the economic value of reducing AF ablation operative times using DES [34]. However, this study differed from ours conducted in China. Kowalski et al. did not include a hospitalization process [34], which may have been a choice in the model design or because AF ablations in their study were day surgeries. In addition, ablation procedures were arranged in block schedules such that two ablations were performed in an EP lab each day, with the possibility of one additional procedure [34], placing a hard limit on the number of procedures per day. In our model, accounting for competition of ward beds was important because of the almost 100% utilization of hospital beds in large tertiary hospitals in China [35]. Furthermore, EP procedures in China do not follow a block schedule. Procedures can start as soon as the previous procedure is completed and the EP lab is ready. Therefore, there is more potential to increase the number of procedures by reducing operative time.

Although the DES technique is increasingly used in healthcare research, applying it as a generalized decision-making tool is challenging. First, a DES model based on the detailed practices of one hospital may not be suitable for other settings. In addition, employing a DES requires a large amount of data, and more detailed data increases cost and time [36]. Adapting a DES model to a new setting requires new data [37], and new data is again required if the system undergoes a change [38]. Furthermore, the simulated results of specific actions may differ from actual implementation owing to human variation [39].

In complex systems such as healthcare, a decision-making tool may be more helpful to clarify generic activity patterns instead of focusing excessively on specific processes of one particular site [38]. Therefore, we summarized and abstracted the processes at two different hospitals to build a generalized DES model of the inpatient EP care delivery process. Lowering the complexity of the model would also greatly reduce the effort required to collect data and facilitate model updates if system changes are made. This model does not prescribe specific actions but instead identifies the bottleneck of the care delivery process. Knowing which phase of the process to prioritize, the decision-maker can consider several different options to improve the healthcare delivery process and use the model to understand the expected results of these options. Further, they can also select the most worthwhile option by considering the time and effort involved compared to the expected increase in total discharges.

This study has several limitations. First, the generalizability of this model may be constrained as it was based on data from two tertiary hospitals collected over a span of two months. This model might not fully reflect the care delivery processes in other hospitals within China or in other countries. The input data may not account for potential seasonal or procedural variations. Additional research is needed to validate the applicability of the approach in more diverse settings. In addition, our approach involved testing scenarios where hospitalization time was reduced by a specified percentage. However, depending on the technology or practice applied to improve the workflow, not all patients may benefit; identifying the proportion of affected patients would yield a more robust analysis. Due to the absence of such data currently, we opted to test scenarios with varying degrees of hospitalization stay reduction to characterize the uncertainty surrounding this aspect. Furthermore, we did not include scenarios with reduced variability, although this may also impact total discharges. However, we tested several scenarios, and the changes were relatively minor. Moreover, reducing variability without changing the mean in practice is difficult, and therefore these scenarios were not included in the results. Finally, the distributions of the simulated number of discharges in some scenarios are quite wide, implying that even if the simulations are accurate, the implementation results would still be uncertain.

## Conclusion

This study developed a generalized DES model to simulate an EP patient’s hospitalization and treatment at a tertiary hospital in China. Using this model, hospital decision-makers dealing with different resource constraints can identify which phase of the care delivery process to optimize in order to better meet demand for EP treatment. Simulations showed that if ward beds are fully occupied, reducing the length of stay of AF ablation patients by 10–30% resulted in a 1–7% increase in the total number of patients discharged. On the other hand, under the condition of fully occupied EP labs, reducing the operative time of AF ablation patients by 10–30% increased discharges by 3–12%.

## Data Availability

Data used in this study are available for research only. Data requests will be reviewed by the review committee of the First Affiliated Hospital of Zhejiang University.

## Abbreviations

DES: discrete event simulation
EP: electrophysiology
CLS: capacity-limiting scenario
AF: atrial fibrillation
FAHZU: First Affiliated Hospital of Zhejiang University
TEP patients: target electrophysiology patients
CLEP patients: catheter lab electrophysiology patients
NEP: non- electrophysiology patients
PVC: premature ventricular complex
SVT: supraventricular tachycardia
AFlutter: atrial flutter
PAC: premature atrial contraction
AT: atrial tachycardia
GP: ganglionated plexi
PFO: patent foramen ovale
ICD: implantable cardioverter defibrillator
PM: pacemaker
CRT: cardiac resynchronization therapy
